# Prognostic benefit of catheter ablation of atrial fibrillation in heart failure: An updated meta‐analysis of randomized controlled trials

**DOI:** 10.1002/joa3.12812

**Published:** 2023-01-17

**Authors:** Sohaib A. Virk, Karice Hyun, David Brieger, Raymond W. Sy

**Affiliations:** ^1^ Department of Cardiology Concord Repatriation General Hospital Concord New South Wales Australia; ^2^ Westmead Applied Research Centre University of Sydney New South Wales Australia; ^3^ Faculty of Medicine and Health The University of Sydney Sydney New South Wales Australia

**Keywords:** atrial fibrillation, catheter ablation, heart failure, meta‐analysis

## Abstract

**Background:**

The prognostic role of catheter ablation of atrial fibrillation (AF) in patients with heart failure (HF) remains uncertain, with guideline recommendations largely based on a single trial. We conducted a meta‐analysis of randomized controlled trials (RCTs) assessing the prognostic impact of AF ablation in patients with HF.

**Methods:**

Electronic databases were searched for RCTs comparing ‘AF ablation’ versus ‘other care’ (medical therapy and/or atrioventricular node ablation with pacing) in patients with HF. Primary endpoints were ≥1‐year mortality, HF hospitalization and change in left ventricular ejection fraction (LVEF). Meta‐analyses were performed using random‐effects modelling.

**Results:**

Nine RCTs (*n* = 1462) met inclusion criteria. Compared to ‘other care’, AF ablation significantly reduced ≥1‐year mortality (relative risk [RR] 0.65; 95% confidence intervals [CI], 0.49–0.87) and HF hospitalization (RR 0.64; 95% CI, 0.51–0.81). AF ablation demonstrated significantly greater improvement in LVEF (mean difference [MD] 5.4; 95% CI, 4.4–6.4), 6‐min walk test distance (MD 21.5 meters; 95% CI, 4.6–38.4) and quality of life as measured by Minnesota Living with Heart Failure Questionnaire score (MD 7.2; 95% CI, 2.8–11.7). Meta‐regression analyses showed the beneficial impact of AF ablation on LVEF was significantly blunted by higher prevalence of ischaemic cardiomyopathy.

**Conclusions:**

Our meta‐analysis demonstrates AF ablation is superior to ‘other care’ in improving mortality, HF hospitalization, LVEF and quality of life in patients with HF. However, the highly selected study populations in included RCTs and effect modification mediated by etiology of HF suggests these benefits do not uniformly apply across the HF population.

## Graphical abstract

This meta‐analysis of 9 RCTs demonstrates AF ablation is superior to ‘other care’ in improving mortality, HF hospitalization, LVEF and quality of life in patients with HF. However, the highly selected study populations in included RCTs and effect modification mediated by etiology of HF suggests these benefits do not uniformly apply across the HF population.

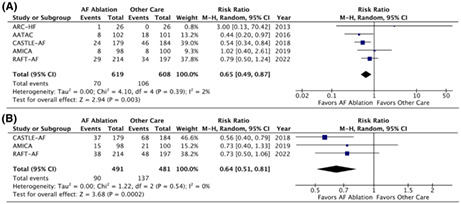

## 1 INTRODUCTION

Atrial fibrillation (AF) and heart failure (HF) are growing epidemics that share common cardiovascular risk factors and frequently co‐exist.^1^ The pathophysiology of AF and HF are intertwined as both facilitate the development and aggravate the course of each other. In patients with HF, the rapid and asynchronous myocardial contraction associated with AF contributes to worsening left ventricular dysfunction, leading to increased risk of HF‐associated hospitalizations and mortality.^2^ On the other hand, HF itself causes increased filling pressures and left atrial (LA) remodelling, thereby predisposing to the development and perpetuation of AF.^3^

Catheter ablation (CA) is an established therapeutic strategy to improve symptoms in patients with AF, demonstrating superior maintenance of sinus rhythm and greater improvement in quality of life compared to anti‐arrhythmic drug (AAD) therapy.^4^, ^5^, ^6^ In the HF population, AF ablation may also have a role beyond symptom control. International guidelines currently provide a class IIA or IIB recommendation for AF ablation in selected patients with HF to “potentially” lower mortality and reduce HF hospitalization.^7^, ^8^ These recommendations were made on the basis of “level B" evidence as they were largely informed by data from a single randomized trial (CASTLE‐AF).^9^ Although several meta‐analyses have assessed the role of AF ablation in patients with HF, results for endpoints of mortality and HF hospitalization were also almost entirely driven by the CASTLE‐AF trial.^10^, ^11^ However, further randomized controlled trials (RCTs) have since been performed assessing the impact of AF ablation on mortality and HF hospitalization in patients with left ventricular dysfunction.^12^, ^13^

Therefore, the primary aim of the present meta‐analysis was to provide an improved level of evidence for determining whether AF ablation has prognostic benefits in patients with HF, based on pooled analysis of randomized data for the endpoints of mortality and HF hospitalization.

## 2 METHODS

### 2.1 Search strategy and study selection

This systematic review and meta‐analysis was conducted in accordance with the Preferred Reporting Items for Systematic Reviews and Meta‐Analyses (PRISMA) guidelines.^14^ Electronic searches were performed using Medline, Embase and Cochrane Central Register of Controlled Trials (CENTRAL) from their dates of inception to March 2022. The search terms “atrial fibrillation” AND “catheter ablation OR pulmonary vein isolation” AND “heart failure OR left ventricular dysfunction” were combined as both keywords and medical subject heading terms. This was supplemented by hand searching the reference lists of key reviews and all potentially relevant studies.

Two reviewers independently screened the title and abstract of records identified in the search. Full‐text publications were subsequently reviewed separately if either reviewer considered the manuscript as being potentially eligible. Disagreements regarding final study inclusion were resolved by discussion with a third investigator.

### 2.2 Eligibility criteria

Eligible studies were RCTs reporting clinical outcomes of ‘AF ablation’ versus ‘other care’ in patients with AF and left ventricular dysfunction. ‘AF ablation’ was defined as pulmonary vein isolation with or without additional substrate modification. ‘Other care’ was defined as medical therapy (rate and/or rhythm control) or atrioventricular node (AVN) ablation with pacing. RCTs reporting post‐hoc subgroup analyses by HF status were only included if trial patients had been stratified by HF status during randomization. To be eligible, studies were required to have a minimum follow‐up duration of 6 months. Non‐English publications, conference abstracts and review articles were excluded. If institutions published duplicate studies with accumulating numbers of patients or increased lengths of follow‐up, only the most complete reports were included.

### 2.3 Data extraction and quality assessment

All data were independently extracted from text, tables and figures by two investigators. Discrepancies were resolved by discussion and consensus. For each study, the following information was extracted: study period, comparator group, inclusion criteria, follow‐up duration, baseline clinical and echocardiographic characteristics, procedural details, endpoint assessment methods and outcome measures.

For all included trials, risk of bias was assessed according to the updated Cochrane Collaboration Risk‐of‐Bias Tool version 2.^15^ Five domains of bias were evaluated: (i) randomization process, (ii) deviations from study protocol, (iii) missing outcome data, (iv) outcome measurement, and (v) selective reporting of results.

### 2.4 Endpoints

The primary outcomes of interest were all‐cause mortality at ≥1‐year, HF hospitalization at follow‐up, and change in left ventricular ejection fraction (LVEF). When studies measured LVEF using multiple imaging modalities, the measurement that was specified as the primary endpoint by trial authors was used for quantitative analysis. Secondary outcomes of interest included: 6‐minute walk test (6 MWT) distance, quality of life (QoL) as measured by the Minnesota Living with Heart Failure Questionnaire (MLHFQ),^16^ stroke at follow‐up, change in maximal oxygen consumption at peak exercise (VO_2_ max), and incidence of peri‐procedural complications (stroke, cardiac tamponade, vascular access complications, pulmonary vein stenosis, atrioesophageal fistula and death). Peri‐procedural complications were defined as those occurring within 30 days following ablation or during the same hospitalization.

### 2.5 Statistical analysis

The mean difference (MD) or relative risk (RR) were used as summary statistics, and reported with 95% confidence intervals (CI). Meta‐analyses were performed using random‐effects models to take into account the anticipated clinical and methodological diversity between studies. The Tau^2^ statistic was used to assess the between study variance and the *I* ^2^ statistic was used to estimate the percentage of total variation across studies due to heterogeneity rather than chance. Cochran's *Q* test was used to test homogeneity, with *p* value <0.1 indicating significant heterogeneity. For meta‐analysis of continuous data, values presented as median and interquartile range were converted to mean and standard deviation using the quantile method previously described by Wan et al.^17^

Meta‐regression analyses were performed to explore potential heterogeneity with the following continuous moderator variables individually assessed for significance: publication year, mean age, proportion of male participants, duration of AF, baseline LVEF, baseline LA diameter, proportion of participants with New York Heart Association (NYHA) III/IV at baseline, and proportion of participants with ischemic cardiomyopathy. Study‐level covariate values represented an average of the two treatment arms in each trial. Pre‐specified subgroup analyses were performed on the basis of control group (rate control versus rhythm control) and AF type (paroxysmal versus persistent). Sensitivity analyses were conducted, excluding: (i) patients with HF with preserved ejection fraction (HFpEF), and (ii) patients who underwent AVN ablation with pacing.

Publication bias was assessed using funnel plots comparing log of point estimates with their standard error. Egger's linear regression method and Begg's rank correlation test were used to detect funnel plot asymmetry.^18^, ^19^ Statistical analysis was conducted with Review Manager Version 5.3.5 (Cochrane Collaboration, Oxford, UK) and Comprehensive Meta‐Analysis v3.0 (Biostat Inc).

## 3 RESULTS

A total of 2591 unique records were identified through the database and bibliographic searches. Of these, 2500 were excluded on the basis of title and abstract content. After screening the full text of the remaining 91 articles, 9 RCTs including a total of 1462 participants were included.^9^, ^12^, ^13^, ^20^, ^21^, ^22^, ^23^, ^24^, ^25^ The study selection process is summarized as a PRISMA flowchart in Supplementary Figure S1.

A summary of study characteristics is displayed in Table 1. In 6 RCTs, AF ablation was compared with a comparator group of rate control that was achieved via medical therapy,^21^, ^22^, ^23^, ^25^ AVN ablation with pacing^20^ or a combination of the two approaches.^13^ In 2 RCTs, the control group received medical therapy for rate and/or rhythm control^9^, ^12^ while one trial specifically compared AF ablation with amiodarone.^24^ Six RCTs exclusively enrolled patients with persistent AF^12^, ^21^, ^22^, ^23^, ^24^, ^25^; in the remaining 3 trials, the proportion of patients with paroxysmal AF ranged from 11%–52%.^9^, ^13^, ^20^ Only one trial included participants with HFpEF.^13^ Median follow‐up duration was 6 months in 3 trials,^20^, ^23^, ^25^ 12 months in 2 trials,^12^, ^22^ 24 months in 1 trial,^24^ and ~37 months in 2 trials.^9^, ^13^

**TABLE 1:** Study characteristics of randomized controlled trials comparing atrial fibrillation ablation and other care in patients with heart failure

	PABA‐CHF 2008	McDonald et al 2011	ARC‐AF 2013	CAMTAF 2014	AATAC 2016	CAMERA‐MRI 2017	CASTLE‐AF 2018	AMICA 2019	RAFT‐AF 2022
Comparison arms	AF ablation vs rate control (AVN ablation with pacing)	AF ablation vs rate control (medical therapy)	AF ablation vs rate control (medical therapy)	AF ablation vs rate control (medical therapy)	AF ablation vs amiodarone	AF ablation vs rate control (medical therapy)	AF ablation vs rate or rhythm control (medical therapy)	AF ablation vs rate or rhythm control (medical therapy)	AF ablation vs rate control (medical therapy or AVN ablation with pacing)
Inclusion	Persistent or paroxysmal AF, NYHA II‐III HF despite optimal HF therapy, LVEF ≤40%	Persistent AF, NYHA II‐IV HF despite optimal HF therapy for ≥3 months, LVEF <35%, absence of contraindication to CMR	Persistent AF, NYHA II‐IV HF despite optimal HF therapy, LVEF ≤35%	Persistent AF, NYHA II‐IV HF, LVEF <50%	Persistent AF, NYHA II‐III HF, LVEF≤40%, dual chamber ICD or CRT‐D	Persistent AF, NYHA II‐IV HF, LVEF ≤45%, no significant CAD or other cause of LV dysfunction	Persistent or paroxysmal AF, NYHA II‐IV HF; LVEF ≤35%	Persistent AF, NYHA II‐III HF, LVEF ≤35%	Persistent or high burden paroxysmal AF, NYHA II‐III, elevated NT‐proBNP
Primary endpoint	Composite of: change in LVEF, 6 MWT distance and QoL (MLHFQ score) from baseline to 6 months	Change in LVEF from baseline to 6 months (on CMR)	Change in VO_2_ at 12 months	Change in LVEF from baseline to 6 months	Freedom from recurrent atrial tachyarrhythmia off AAD at follow‐up	Change in LVEF from baseline to 6 months (on CMR)	Death from any cause or worsening of heart failure that led to an overnight hospitalization	Change in LVEF from baseline to 12 months	Composite of time to death or HF event
Secondary endpoints	Freedom from AF and change in LA diameter at 6 months	Change in RVEF, LVESV, LVEDV, LA diameter, BNP, 6 MWT distance and QoL (KCCQ, MLHFQ and SF‐36 scores).	Change in QoL (MWLHFQ score), BNP, and 6 MWT distance	Percentage reduction in LVESV, change in VO_2_ max, BNP, NYHA class, and QoL (MLHFQ and SF‐36 scores)	Procedural complications, all‐cause mortality, AF and HF‐related unplanned hospitalizations during follow‐up, change in LVEF, 6 MWT distance, and QoL (MLHFQ score)	Change in: cardiac chamber dimensions (on CMR), NYHA class, BNP level, 6 MWT distance, QoL (SF‐36 scores); AF recurrence; AF burden; procedural complications.	Cardiovascular death, stroke, all hospitalizations, hospitalizations for cardiovascular disease; AF‐free survival; procedural complications	Change in: 6 MWT distance, QoL (MLHFQ score), BNP; AF burden, procedural complications, all‐cause mortality	Change in LVEF, NT‐proBNP, 6 MWT distance, and QoL (MLHFQ score) at 12 and 24 months
LV function modality	TTE	CMR and radionucleotide ventriculography	TTE	TTE	TTE	CMR	TTE	TTE	TTE
Follow‐up duration^a^ (months)	6^M^	8.4 ± 2.5	12^M^	6–12 (range)	24^M^	6^M^	37.5 ± 19.0	11.9 ± 2.4	37.4 [24.7–53.7]
Definition of recurrent AF	AF lasting >30 s	NR	Any atrial arrhythmia lasting >30 s	AF or atrial tachycardia lasting >30 s	AF, atrial flutter or atrial tachycardia lasting >30 s	AF or atrial tachycardia lasting >30 s	Any atrial arrhythmia lasting >30 s	NR	AF documented on 12‐lead ECG
Blanking period (months)	2	3	2	3	3	1	3	3	1–1.5
Heart rhythm monitoring protocol	Weekly transmissions via loop event monitor, formal visit at 6 months	Holter at baseline and 6 months	ECG rhythm documentation at 6 to 8 weeks and all follow‐ups 48‐h Holter at 3, 6 and 12 months.	Holter at 1, 3 and 6 months	Remote interrogation of implantable device at 3, 6, 12, and 24 months	Continuous rhythm monitoring via implanted loop recorder plus interrogations at 6 weeks, 3 and 6 months	Device home monitoring with scheduled device interrogations at 3, 6, 12, 24, 36, 48, and 60 months	Daily smartphone ECG recordings 12‐lead ECG at 1, 3, 6 and 12 months	ECG at 2, 4, 6 months and then 6‐monthly

Abbreviations: 6 MWT, 6‐min walk test; AAD, anti‐arrhythmic drugs; AF, atrial fibrillation; AVN, atrioventricular node; CAD, coronary artery disease; CMR, cardiac magnetic resonance; CRT‐D, cardiac resynchronization therapy‐defibrillator; ECG, electrocardiogram; HF, heart failure; ICD, implantable cardioverter‐defibrillator; KCCQ, Kansas City Cardiomyopathy Questionnaire; LA, left atrial; LV, left ventricular; LVEDV, left ventricular end diastolic volume; LVEF, left ventricular ejection fraction; LVESV, left ventricular end systolic volume; M, median; MLHFQ, Minnesota Living with Heart Failure Questionnaire; NT‐proBNP, N‐terminal pro brain natriuretic peptide; NYHA, New York Heart Association; QoL, quality of life; RVEF, right ventricular ejection fraction; SF, 36‐short form 36, TTE, transthoracic echocardiography; VO_2_, peak oxygen consumption.

a Data presented as mean ± standard deviation or median [interquartile range], unless otherwise indicated.

Across all RCTs, the proportion of patients screened but excluded prior to randomization ranged from 49%–89%. Included participants had a weighted mean age of 63.8 years and 82% were male. Mean baseline LVEF varied from 17.7%–40.6% across studies. The proportion of patients with NYHA III/IV symptoms ranged from 12.5%–60.7% and the aetiology of heart failure was ischemic in 0%–70.4% of participants across trials. Mean duration of AF in individual studies ranged from 21 to 53 months. With regards to medical therapy for HF, 88% of total participants were on angiotensin‐converting enzyme inhibitors/angiotensin II receptor blockers, 91% on beta‐blockers and 54% on mineralocorticoid receptor antagonists. A complete summary of the baseline clinical and echocardiographic characteristics of trial participants is provided in Table 2.

**TABLE 2:** Summary of baseline participant characteristics in randomized controlled trials comparing atrial fibrillation ablation and other care in patients with heart failure

	PABA‐CHF 2008		McDonald et al 2011		ARC‐HF 2013		CAMTAF 2014		AATAC 2016		CAMERA‐MRI 2017		CASTLE‐AF 2018		AMICA 2019		RAFT‐AF 2022	
	AFA	OC	AFA	OC	AFA	OC	AFA	OC	AFA	OC	AFA	OC	AFA	OC	AFA	OC	AFA	OC
Number screened	177		366		101		390		866		301		3013		NR		NR	
Total study number	81		41		52		50		203		66		363		195 (140 with full data for analysis)		411	
Group Numbers	41	40	22	19	26	26	26	24	102	101	33	33	179	184	68	72	214	197
Age, yrs	60 ± 8	61 ± 8	62.3 ± 6.7	64.4 ± 8.3	64 ± 10	62 ± 9	55 ± 12	60 ± 10	62 ± 10	60 ± 11	59 ± 11	62 ± 9	64 [56–71]	64 [56–74]	65 ± 8	65 ± 8	65.9 ± 8.6	67.5 ± 8.0
Male, *n* (%)	39 (41)	35 (88)	17 (77)	15 (79)	21 (81)	24 (92)	25 (96)	23 (96)	77 (75)	74 (73)	31 (94)	29 (88)	156 (87)	155 (84)	60 (88)	66 (92)	157 (73)	148 (75)
Paroxysmal AF, *n* (%)	20 (49)	22 (55)	0		0		0		0		0		54 (30)	64 (35)	0		19 (9)	11 (6)
Persistent AF, n (%)	21 (51)	18 (45)	41 (100)		52 (100)		50 (100)		203 (100)		66 (100)		125 (70)	120 (65)	195 (100)		195 (91)	186 (94)
Long standing (>1 year), *n* (%)	NR	NR	NR		NR		48/50 (96)		NR		49/66 (74)		106/363 (29)		33/140 (24)		96/411 (23)	
Continuous AF (median; months)	NR	NR	NR		23.5^M^		24^M^		8.5^M^		22^M^		NR		NR		NR	
Time since AF diagnosis, months	48 ± 28.8	46.8 ± 33.6	44 ± 36.5	64 ± 47.6	23 ± 22	51 ± 76	23 [17–33]	24 [12–48]	NR	NR	NR	NR	NR	NR	NR	NR	14.5 [7–36]	15 [6–48]
Hypertension, *n* (%)	NR	NR	14 (64)	11 (58)	NR	NR	8 (31)	8 (33)	46 (45)	48 (48)	13 (39)	12 (36)	129 (72)	136 (74)	56 (82)	55 (76)	140 (65)	132 (67)
Diabetes, *n* (%)	NR	NR	7 (32)	4 (21)	NR	NR	NR	NR	22 (22)	24 (24)	4 (12)	5 (15)	43 (24)	67 (36)	24 (35)	22 (31)	61 (29)	64 (32)
Prior CVA, *n* (%)	NR	NR	2 (9)	2 (11)	NR	NR	NR	NR	NR	NR	2 (6)	0	21 (12)	21 (11)	NR	NR	19 (9)	20 (10)
Ischemic cardiomyopathy, *n* (%)	30 (73)	27 (68)	11 (50)	9 (47)	10 (38)	7 (27)	6 (23)	7(29)	63 (62)	66 (65)	0	0	72 (40)	96 (52)	30 (44)	40 (56)	74 (35)	55 (28)
NYHA III/IV (%)	NR	NR	20 (91)	17 (89)	12 (46)	13 (50)	15 (58)	12 (50)	NR	NR	NR	NR	53/174 (31)	51/179 (28)	40 (59)	45 (62)	70 (33)	66 (34)
*Medical therapy*																		
ACE‐I/ARB	NR	NR	21 (95)	18 (95)	25 (96)	26 (100)	26 (100)	24 (100)	94 (92)	89 (88)	31 (94)	31 (94)	166/177 (94)	164/180 (91)	62 (91)	68 (94)	155 (72)	161 (82)
Beta blocker	NR	NR	18 (82)	18 (95)	24 (92)	24 (92)	26 (100)	24 (100)	78 (76)	81 (80)	32 (97)	32 (97)	165/177 (93)	171/180 (95)	62 (91)	67 (93)	197 (92)	182 (92)
MRA	NR	NR	10 (45)	3 (16)	13 (50)	6 (23)	NR	NR	46 (45)	51 (50)	11 (33)	16 (48)	165/177 (93)	167/180 (93)	44 (65)	48 (67)	51 (24)	53 (27)
Digoxin	NR	NR	12 (55)	9 (47)	16 (62)	12 (46)	NR	NR	NR	NR	NR	NR	NR	NR	20 (29)	21 (29)	55 (26)	65 (33)
Amiodarone	33 (80)	36 (90)	NR	NR	3 (12)	3 (12)	NR	NR	NR	101 (100)	NR	NR	55 (31)	46 (26)	17 (25)	27 (38)	NR	NR
LVEF % at baseline	27 ± 8	29 ± 7	16.1 ± 7.1	19.6 ± 5.5	22 ± 8	25 ± 7	31.8 ± 7.7	33.7 ± 12.1	29 ± 5	30 ± 8	32 ± 9	34 ± 8	33 [25–38]	31.5 [27–37]	27.8 ± 9.5	24.8 ± 8.8	40.9 ± 14.9	40.3 ± 14.7
LA diameter at baseline, mm	49 ± 5	47 ± 6	NR	NR	50 ± 6	46 ± 7	52 ± 11	50 ± 10	47 ± 4.2	48 ± 4.9	48 ± 5.5	47 ± 8.2	48 [45–54]	49.5 [50–55]	50 ± 6	51 ± 5	46.8 ± 5.4	46.1 ± 6.0
No. of AF ablation procedures	1.20		1.23		1.19		1.7 ± 0.7		1.4 ± 0.6		1		1.3 ± 0.5		1.16		1.41	
RFA procedure duration (mins)	NR		205		333 ± 61		NR		168 ± 22		200 ± 47		NR		157 ± 47		210 ± 60	

*Note*: Continuous data presented as mean ± standard deviation or median [interquartile range], unless otherwise indicated.

Abbreviations: ACE‐I, angiotensin converting enzyme inhibitor; AF, atrial fibrillation; AFA, atrial fibrillation ablation; ARB, angiotensin II receptor blocker; CA, catheter ablation; CAD, coronary artery disease; CVA, cerebrovascular accident; HF, heart failure; LA, left atrial; LVEF, left ventricular ejection fraction; MRA, mineralocorticoid receptor antagonist; NYHA, New York Health Association; NR, not reported; OC, other care; RFA, radiofrequency ablation.

Ablation strategy varied within and between studies (Table 3). All studies performed pulmonary vein isolation with the majority also reporting additional linear ablation (e.g. left atrial roof and/or mitral isthmus lines) and targeting of complex fractionated atrial electrograms at the discretion of the operator.

**TABLE 3:** Summary of ablation strategies employed in randomized controlled trials assessing atrial fibrillation ablation in patients with heart failure

Study	PVI	Additional linear ablation^a^	Posterior wall isolation	SVC isolation	CFAE ablation	Elimination of AF triggers
PABA‐CHF 2008	√	√			√	
MacDonald 2011	√	√			√	
ARC‐HF 2013	√	√			√	
CAMTAF 2014	√	√			√	
AATAC 2016	√	√	√	√	√	√
CAMERA‐MRI 2017	√		√			
CASTLE‐AF 2018	√	√	√	√	√	√
AMICA 2019	√	√			√	
RAFT‐AF 2022	√	√	√		√	

Abbreviations: AF, atrial fibrillation; CFAE, complex fractionated atrial electrograms; PVI, pulmonary vein isolation; SVC, superior vena cava.

^a^ Includes ablation of the left atrial roof, mitral isthmus and/or cavotricuspid isthmus.

### 3.1 Study quality

All included trials employed robust randomization and allocation concealment procedures. There was high risk of bias from protocol deviations in one study due to post‐randomization changes to inclusion criteria and significant rates of cross‐over between treatment arms.^9^ One study had significant missing data for the endpoint of LVEF change.^12^ All studies had low risk of measurement bias for endpoint of LVEF change with reporting by blinded assessors. However, in four trials, there was high risk of measurement bias due to unblinded assessment of subjective secondary endpoints.^12^, ^23^, ^24^, ^25^ Two trials were terminated early due to presumed futility,^12^, ^13^ and one trial performed its final analysis prior to reaching pre‐specified enrolment and event targets.^9^ A complete summary of the risk of bias assessment for included trials is displayed in Supplementary Table S1.

### 3.2 Procedural outcomes

Overall, in the 740 patients across included trials who underwent AF ablation, the pooled incidence of AF recurrence requiring repeat ablation was 24% per year (95% CI, 17.0–35.7; *I* ^2^ = 66%). At last follow up, pooled rate of freedom from AF after one or more procedures was 76.3% (95% CI, 66.8–83.8; *I* ^2^ = 81%). Patients underwent multiple procedures in 8 trials, with the mean number of ablations ranging from 1.16–1.7. Compared to other care, AF ablation significantly reduced the risk of recurrent AF at follow‐up (RR 0.29; 95% CI, 0.19–0.44; Tau^2^ = 0.30; *I* ^2^ = 87%; *p* < 0.001).

Major peri‐procedural complications included stroke (1.9%; 95% CI, 1.0–3.5), cardiac tamponade (2.5%; 95% CI, 1.3–4.7), pulmonary vein stenosis (1.4%; 95% CI, 0.7–3.1) and vascular access complications (2.3%; 95% CI, 1.3–4.0). Two trials each reported a single case of atrio‐oesophageal fistula.^12^, ^13^ There were two peri‐procedural deaths reported.

### 3.3 Primary outcomes

**FIGURE 1:**
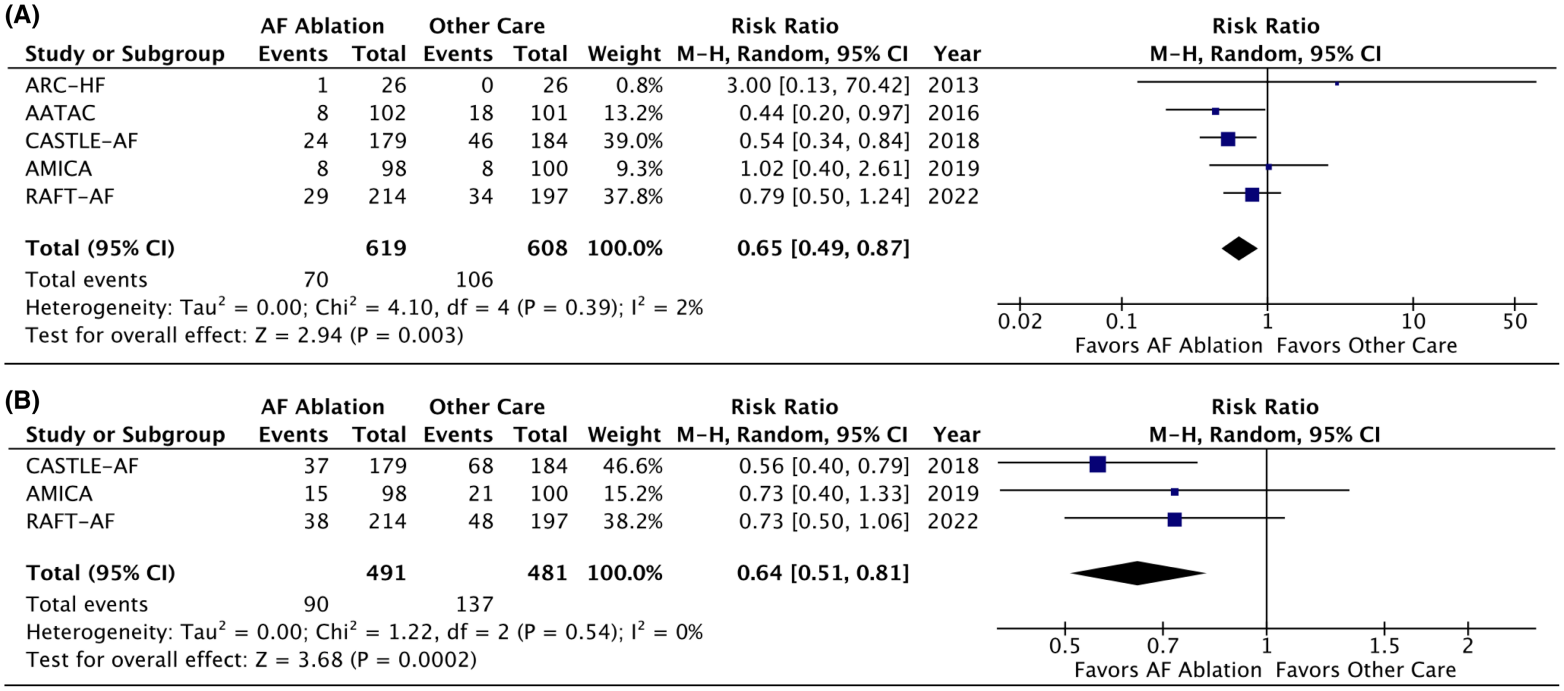
Forest plot displaying relative risk (RR) for: (A) mid‐term mortality, and (B) heart failure hospitalisation, in patients with heart failure and atrial fibrillation (AF) undergoing AF ablation versus other care.

Five RCTs involving 1227 participants reported on all‐cause mortality at ≥1 year, demonstrating a significant mortality benefit with AF ablation (RR 0.65; 95% CI, 0.49–0.87; Tau^2^ = 0; *I* ^2^ = 2%; *p* = 0.003; Figure 1A). In 3 RCTs involving 972 participants, HF hospitalizations at follow‐up were significantly reduced following AF ablation compared to other care (RR 0.64; 95% CI, 0.51–0.81; Tau^2^ = 0; *I* ^2^ = 0%; *p* < 0.001; Figure 1B).

**FIGURE 2:**
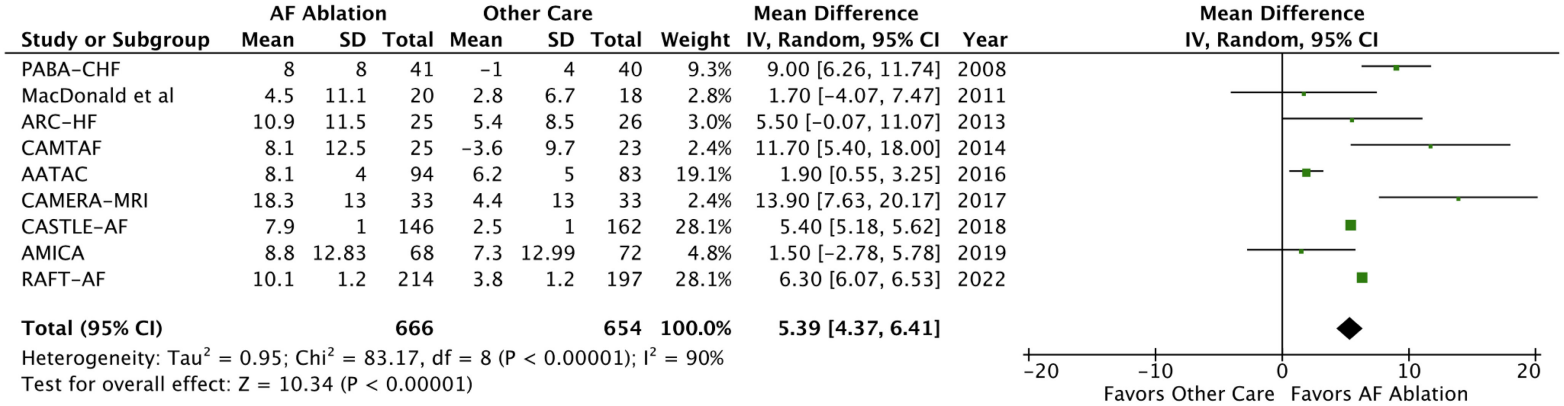
Forest plot displaying mean difference (MD) in left ventricular ejection fraction (LVEF) change in patients with heart failure and atrial fibrillation (AF) undergoing AF ablation versus other care.

In 9 RCTs reporting echocardiographic data on 1320 participants, AF ablation demonstrated significantly greater improvement in LVEF (MD 5.39%; 95% CI, 4.37–6.41; Tau^2^ = 0.95; *I* ^2^ = 90%; *p* < 0.001; Figure 2). Repeating the analysis using only LVEF values derived from transthoracic echocardiography did not impact the significant of this result; nor did it ameliorate heterogeneity (MD 5.38%; 95% CI, 4.38–6.38; Tau^2^ = 0.88; *I* ^2^ = 91%; *p* < 0.001).

### 3.4 Secondary outcomes

**FIGURE 3:**
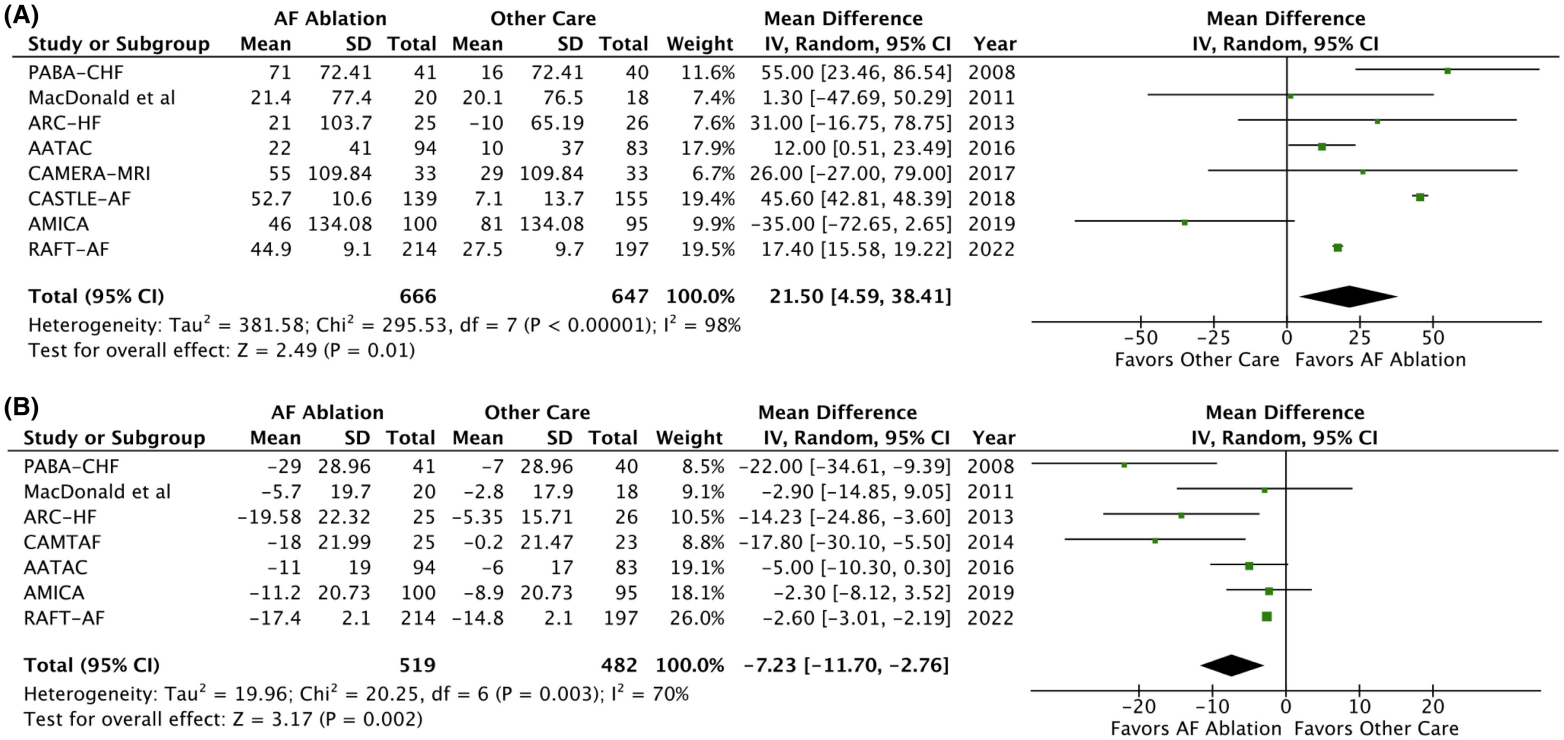
Forest plot displaying mean difference (MD) in: (A) 6‐min walk test distance and (B) Minnesota living with heart failure questionnaire scores in patients with heart failure and atrial fibrillation (AF) undergoing AF ablation versus other care.

In 8 RCTs reporting on 1313 participants, AF ablation was associated with significantly greater increase in 6 MWT distance (MD 21.50 meters; 95% CI, 4.59–38.41; Tau^2^ = 381.58; *I* ^2^ = 98%; *p* = 0.01; Figure 3A). In 7 RCTs reporting on 1001 participants, patients who underwent AF ablation had significantly greater improvement in QoL as demonstrated by larger reduction in MLHFQ score (MD 7.23; 95% CI, 2.76–11.70; Tau^2^ = 19.96; *I* ^2^ = 70%; *p* = 0.002; Figure 3B). In 2 RCTs reporting on 774 participants,^9^, ^13^ AF ablation did not significantly reduce the incidence of stroke at follow‐up (RR 0.62; 95% CI, 0.28–1.37; *I* ^2^ = 0%; *p* = 0.24). In 2 RCTs reporting on 102 participants,^22^, ^23^ AF ablation was associated with significantly greater increase in VO_2_ max (MD 3.16; 95% CI, 1.05–5.27; Tau^2^ = 0; *I* ^2^ = 0%; *p* = 0.003).

### 3.5 Subgroup analyses

Subgroup analyses were performed according to AF type. In the 6 RCTs (*n* = 520) that exclusively enrolled patients with persistent AF,^12^, ^21^, ^22^, ^23^, ^24^, ^25^ ablation demonstrated significantly greater improvement in LVEF (MD 5.47%; 95% CI, 1.66–9.27; Tau^2^ = 16.12; *I* ^2^ = 78%; *p* = 0.005) and QoL (MD 7.02; 95% CI, 1.81–12.22; Tau^2^ = 16.16; *I* ^2^ = 49%; *p* = 0.008) compared to other care. However, there was no significant benefit with regards to 6 MWT distance (MD 6.02 meters; 95% CI, −14.50 to 26.54; Tau^2^ = 218.63; *I* ^2^ = 41%; *p* = 0.57) or all‐cause mortality at ≥1 year (RR 0.70; 95% CI, 0.33–1.50; Tau^2^ = 0.30; *I* ^2^ = 27%; *p* = 0.36). There was insufficient data to enable separate analyses for the paroxysmal AF population.

Further subgroup analysis was performed according to type of comparator group (i.e. rate or rhythm control). In the 6 RCTs (*n* = 695) that compared AF ablation with rate control,^13^, ^20^, ^21^, ^22^, ^23^, ^25^ ablation was superior with regards to improving LVEF (MD 7.65; 95% CI, 5.15–10.16; Tau^2^ = 5.16; *I* ^2^ = 66%; *p* < 0.001), 6 MWT distance (MD 24.86 meters; 95% CI, 8.19–41.53; Tau^2^ = 133.74; *I* ^2^ = 36%, *p* = 0.003) and QoL (MD 10.96; 95% CI, 2.33–19.59; Tau^2^ = 70.34; *I* ^2^ = 79%, *p* = 0.01). Only 2 of the RCTs (*n* = 463) comparing AF ablation versus rate control reported on mid‐term mortality and the pooled result was not significant (RR 0.81; 95% CI, 0.51–1.27; Tau^2^ = 0; *I* ^2^ = 0%, *p* = 0.35).^13^, ^22^

AF ablation was compared with pharmacological rate control in 4 RCTs.^21^, ^22^, ^23^, ^25^ There remained significantly greater improvement in LVEF (MD 8.07; 95% CI, 2.60–13.53; *I* ^2^ = 70%; *p* = 0.004) and QoL (MD 11.70; 95% CI, 3.17–20.23; *I* ^2^ = 38%; *p* = 0.007), but not 6 MWT distance (MD 19.31; 95% CI, −9.42 to 48.05; *I* ^2^ = 0%; *p* = 0.19).

### 3.6 Sensitivity analyses

Sensitivity analyses were performed by excluding two RCTs in which control participants underwent AVN ablation with pacing.^13^, ^20^ AF ablation was superior to medical therapy with regards to LVEF improvement (MD 5.08; 95% CI, 2.63–7.53; Tau^2^ = 6.51; *I* ^2^ = 85%; *p* < 0.001), QoL (MD 7.02; 95% CI, 1.81–12.22; Tau^2^ = 16.06; *I* ^2^ = 49%, *p* = 0.008), mid‐term mortality (RR 0.58; 95% CI, 0.40–0.83; Tau^2^ = 0; *I* ^2^ = 1%; *p* = 0.003) and HF hospitalization (RR 0.60; 95% CI, 0.44–0.80; Tau^2^ = 0; *I* ^2^ = 0%; *p* < 0.001). However, the difference in 6 MWT distance was not significant (MD 15.50 meters; 95% CI, −9.52 to 40.52; Tau^2^ = 671.70; *I* ^2^ = 90%; *p* = 0.22).

Further sensitivity analyses were performed by excluding 171 patients from 1 RCT that enrolled a subset of participants with HFpEF.^13^ In the remaining 1291 patients with HF with reduced ejection fraction (HFrEF), AF ablation remained superior to other care with regards to improvement in LVEF (MD 5.49; 95% CI, 4.31–6.68; Tau^2^ = 1.43; *I* ^2^ = 90%; *p* < 0.001) and QoL (MD 7.26; 95% CI, 2.39–12.14; Tau^2^ = 25.85; *I* ^*2*^ = 75%; *p* = 0.003), but the difference in 6 MWT distance was not statistically significant (MD 18.56 meters; 95% CI, −3.36 to 40.47; Tau^2^ = 745.90; *I* ^2^ = 98%; *p* = 0.10). Data for endpoints of mortality and HF hospitalization was not separately reported for the subset of patients with HFpEF.

### 3.7 Meta‐regression

**FIGURE 4:**
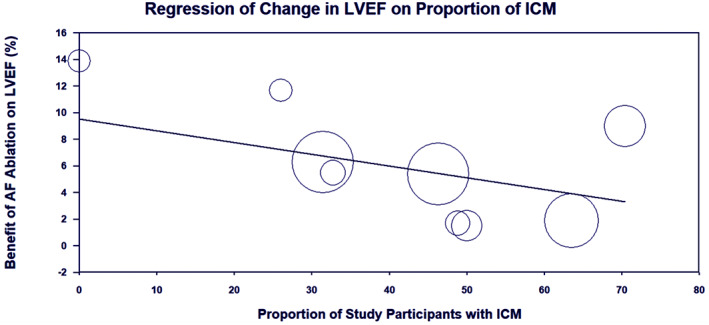
Scatterplot displaying regression of change in left ventricular ejection fraction (LVEF) on prevalence of ischaemic cardiomyopathy (CM) in randomized controlled trials comparing atrial fibrillation (AF) ablation versus other care in patients with AF and heart failur.

Meta‐regression analyses were performed for the endpoint of LVEF change to explore potential sources of heterogeneity. The proportion of participants with ischaemic cardiomyopathy was a significant moderator; the beneficial impact of AF ablation on LVEF was reduced in studies with higher prevalence of ischaemic cardiomyopathy (coefficient − 0.1; 95% CI, −0.16 to −0.02; *p* = 0.02; Figure 4). Publication year (*p* = 0.42), proportion of male participants (*p* = 0.06), mean age (*p* = 0.30), proportion of participants with NYHA III/IV symptoms (*p* = 0.16), baseline LVEF (*p* = 0.16) and baseline LA diameter (*p* = 0.88) were not found to be significant moderators (Supplementary Table S2). There was insufficient number of studies to enable meta‐regression analyses for other endpoints.

### 3.8 Publication bias

There was no evidence of publication bias for the endpoint of change in LVEF using Egger's linear regression method (*p* = 0.87) or Begg's rank correlation test (*p* = 0.35; Supplementary Figure S2). Publication bias could not be assessed for other endpoints due to insufficient number of trials.

## 4 DISCUSSION

The present meta‐analysis demonstrates that AF ablation in patients with heart failure is associated with significantly greater improvements in mid‐term mortality, HF hospitalization, systolic left ventricular function, exercise capacity and quality of life, compared to ‘other care’. Previous meta‐analyses on this topic have also reported improvement in echocardiographic and functional outcomes following AF ablation.^10^, ^11^ However, prior analysis of mid‐term mortality was limited to ~600 patients (compared with 1227 included in the present study) and analysis of HF hospitalization limited to ~400 patients (compared with 972 included in the present study). Furthermore, in prior meta‐analyses, the pooled estimates for these harder clinical endpoints were virtually identical to the CASTLE‐AF trial as that study provided ~75% of weighting for mortality and > 95% weighting for HF hospitalization.^9^ Thus, by incorporating data from more recent large RCTs, the findings of the present meta‐analysis are based on a much greater number of patients and are not dependent on a single trial, thereby providing a more robust affirmation of the prognostic benefits of AF ablation.

In the present meta‐analysis, AF ablation resulted in a 35% risk reduction in mortality at ≥1‐year, compared to ‘other care’. Although there was low statistical heterogeneity (Tau^2^ = 0, *I* ^2^ = 2%) for this endpoint, the point estimate for the AMICA‐AF trial indicated a complete lack of mortality benefit^12^ in stark contrast to the 21% and 46% risk reductions seen in RAFT‐AF and CASTLE‐AF, respectively (Figure 1A).^9^, ^13^ Of note, AMICA‐AF only had a follow‐up duration of 12 months compared with ≥36 months for the other two trials (Table 1). Indeed, the Kaplan–Meier curves for mortality in both CASTLE‐AF and RAFT‐AF showed late divergence with less difference between treatment arms at the 1‐year interval. As such, it is possible that the mortality benefit following AF ablation in HF may only become apparent after an extended follow‐up period.

With regards to possible mechanisms underpinning the prognostic benefits of AF ablation in HF, our meta‐analysis demonstrated patients undergoing AF ablation had 5.4% greater improvement in LVEF and 36% reduction in HF hospitalization as compared to those receiving ‘other care’. The beneficial impact of AF ablation on left ventricular function may be explained by its superiority in maintaining sinus rhythm, which enables regular ventricular filling and synchronous myocardial contraction. This is supported by the lack of significant improvement in LVEF in the study by MacDonald et al, in which the rate of recurrent AF at follow‐up was highest (50%).^21^ However, there was considerable heterogeneity detected for the endpoint of LVEF change in our meta‐analysis (*I* ^2^ = 90%), and this was not completely ameliorated by subgroup and sensitivity analyses accounting for AF type and differences in control groups. In meta‐regression analyses, aetiology of heart failure was found to be the only significant effect modifier; the beneficial impact of AF ablation on LVEF was significantly diminished in studies enrolling a higher proportion of participants with ischemic cardiomyopathy. This may reflect distinct pathophysiologies whereby, in patients with prior myocardial infarction, AF is more likely to be a by‐product of adverse myocardial remodelling rather than a primary contributor to left ventricular dysfunction. More broadly, ischaemic aetiology may be a surrogate marker of myocardial scar as the presence of ventricular late gadolinium enhancement (LGE) on cardiac magnetic resonance (CMR) has been shown to portend poorer response to AF ablation in patients with HF.^25^ In a small observational study of 16 patients with cardiomyopathy that were prospectively selected for AF ablation based on absence of LGE on CMR imaging, an impressive 20% improvement in LVEF was demonstrated at 6 months.^26^ As such, although further prospective data is required, it appears the use of CMR imaging may have an important role in selecting patients with reversible left ventricular dysfunction who are more likely to benefit from AF ablation.

Of note, the vast majority of patients included in our meta‐analysis had HFrEF, with only one trial (RAFT‐AF) enrolling a subset of patients with HFpEF.^13^ Subgroup analysis of the HFpEF population in RAFT‐AF demonstrated no significant benefit of AF ablation with regards to the combined primary endpoint of all‐cause mortality and HF hospitalization. This is discordant with the findings of the HF subgroup analysis of the CABANA (Catheter Ablation Versus Anti‐arrhythmic Drug Therapy for Atrial Fibrillation) trial, which included 778 participants with heart failure.^27^ Although 79% of HF patients in that sub‐study had preserved left ventricular function, catheter ablation demonstrated a significant reduction in all‐cause mortality (HR 0.57; 95% CI, 0.33–0.96) and this result was reproduced in post‐hoc analysis confined to patients with LVEF ≥50%. This discrepancy is likely explained by the different definition of HFpEF employed in these trials. Whereas inclusion in RAFT‐AF required participants to have both NYHA II/III symptoms and elevated N‐terminal pro brain natriuretic peptide levels, the CABANA HF study included patients based on NYHA II symptoms alone. Given AF itself can cause dyspnoea and fatigue, the lack of objective parameters to define HFpEF in CABANA HF may have introduced heterogeneity in the HF cohort. Furthermore, randomization was not stratified by HF status in the CABANA trial and the results were confounded by high rates of cross‐over and protocol deviations.^28^ As such, the prognostic impact of AF ablation in HFpEF remains unclear and randomized trials specifically including patients with objectively defined HFpEF are warranted.

Overall, the findings of the present meta‐analysis should upgrade the level of evidence in support of AF ablation in patients with HF to “Level A" (i.e. ‘data derived from multiple randomised clinical trials, or meta‐analyses’) and inform future guideline recommendations in this area. However, it should remain a point of emphasis that appropriate selection of HF patients for AF ablation is required because trial cohorts included in this meta‐analysis represent a highly selected HF population. This is evidenced by the low proportion of screened participants who underwent randomization in included RCTs, ranging as low as 11%. Indeed, in a recent retrospective analysis of the US database, only 7.8% of patients with AF and HF were found to be eligible for inclusion in the CASTLE‐AF trial.^29^ Compared to trial patients, those who did not meet inclusion criteria were significantly older and had a higher prevalence of major co‐morbidities including hepatic and renal impairment, major bleeding history, cerebrovascular disease and cognitive impairment.^29^ As such, considerable discretion is required from clinicians in choosing appropriate HF patients for AF ablation. To help guide this decision making, further RCTs are required that focus on specific HF sub‐populations such as those with non‐ischaemic cardiomyopathy or absence of LGE on cardiac MRI.

There have been considerable technological advances in catheter ablation of AF over the past decade with the introduction of novel catheter designs, energy source, and ablation indices (including contact force) aimed at improving safety and efficacy. These temporal changes are likely to favour catheter ablation for AF in heart failure.^30^ However, medical therapy for heart failure has also evolved in recent years, specifically with the emergence of angiotensin receptor‐neprilysin inhibitors (ARNIs) and sodium‐glucose cotransporter‐2 (SGLT2) inhibitors.^31^ Trials included in our meta‐analysis did not report the proportion of patients receiving ARNIs and SGLT2 inhibitors as they largely predated guidelines establishing these as pillars of optimal heart failure therapy. In the absence of individual patient data, it remains unclear whether the relative superiority of AF ablation over medical therapy may be attenuated by more widespread adoption of these medications. While a meta‐regression analysis demonstrated that publication year was not a significant co‐variate (Supplementary Table S2), this analysis may fail to capture the nuances in the evolution of both catheter ablation and medical therapy during the study periods.

The present meta‐analysis has several other limitations, which must be considered when interpreting its findings. Firstly, the pooled freedom from AF at follow‐up was 76.3%, which was unexpectedly high given that most included patients had persistent AF. In comparison, real‐world data from registries has found success rates in the range of 50%–60% following catheter ablation for persistent AF.^32^ The duration of continuous AF is a major determinant of long‐term AF recurrence following catheter ablation,^33^ and this was not reported in the majority of included trials (Table 2), thus limiting interpretation of the pooled recurrence rate. Furthermore, as trials included in this meta‐analysis were conducted at high‐volume centres with highly experienced operators, it remains to be seen whether the success rates (and related benefits) of AF ablation demonstrated in this meta‐analysis can be generalized to a broader clinical setting. Secondly, there were clinical and methodological differences between trials with regards to follow‐up duration, use of concurrent therapies, ablation strategy, severity and aetiology of heart failure, AF duration, and the treatment employed in the control arm. Although we attempted to account for this with subgroup and sensitivity analyses as well as meta‐regression, these were limited by the number of included trials and lack of patient‐level data. In particular, our meta‐regression analyses should only be considered exploratory given the significant propensity for spurious findings in this setting.^34^ Lastly, quantitative synthesis of several outcomes (e.g. major procedural complications) was impeded by a lack of standardized endpoint reporting across included trials.

In conclusion, meta‐analysis of randomized data demonstrates that AF ablation is superior to ‘other care’ in improving mid‐term mortality, HF hospitalization, left ventricular systolic function, functional status, and quality of life in patients with co‐existing AF and HF. However, the highly selected study populations in included RCTs and the effect modification mediated by etiology of heart failure in our meta‐regression analyses suggest these benefits do not uniformly apply across the HF population. Further trials assessing the role of AF ablation in specific HF subgroups are required to identify those that will derive most benefit from ablative therapy.

## CONFLICT OF INTEREST

None.

## FUNDING INFORMATION

None.

## ETHICS APPROVAL STATEMENT

Not applicable (meta‐analysis).

## PATIENT CONSENT STATEMENT

Not applicable (meta‐analysis).

## CLINICAL TRIAL REGISTRATION

Not applicable (meta‐analysis).

## Supporting information


Supplemental Figure S1.

Supplemental Figure S2.

Supplemental Table S1.
