# Supplementary figures and images for: Prognostic benefit of catheter ablation of atrial fibrillation in heart failure: An updated meta‐analysis of randomized controlled trials
Source: J Arrhythm. 2023 Jan 17;39(2):129–41. doi: 10.1002/joa3.12812 (PMC10068943; doi:10.1002/joa3.12812)

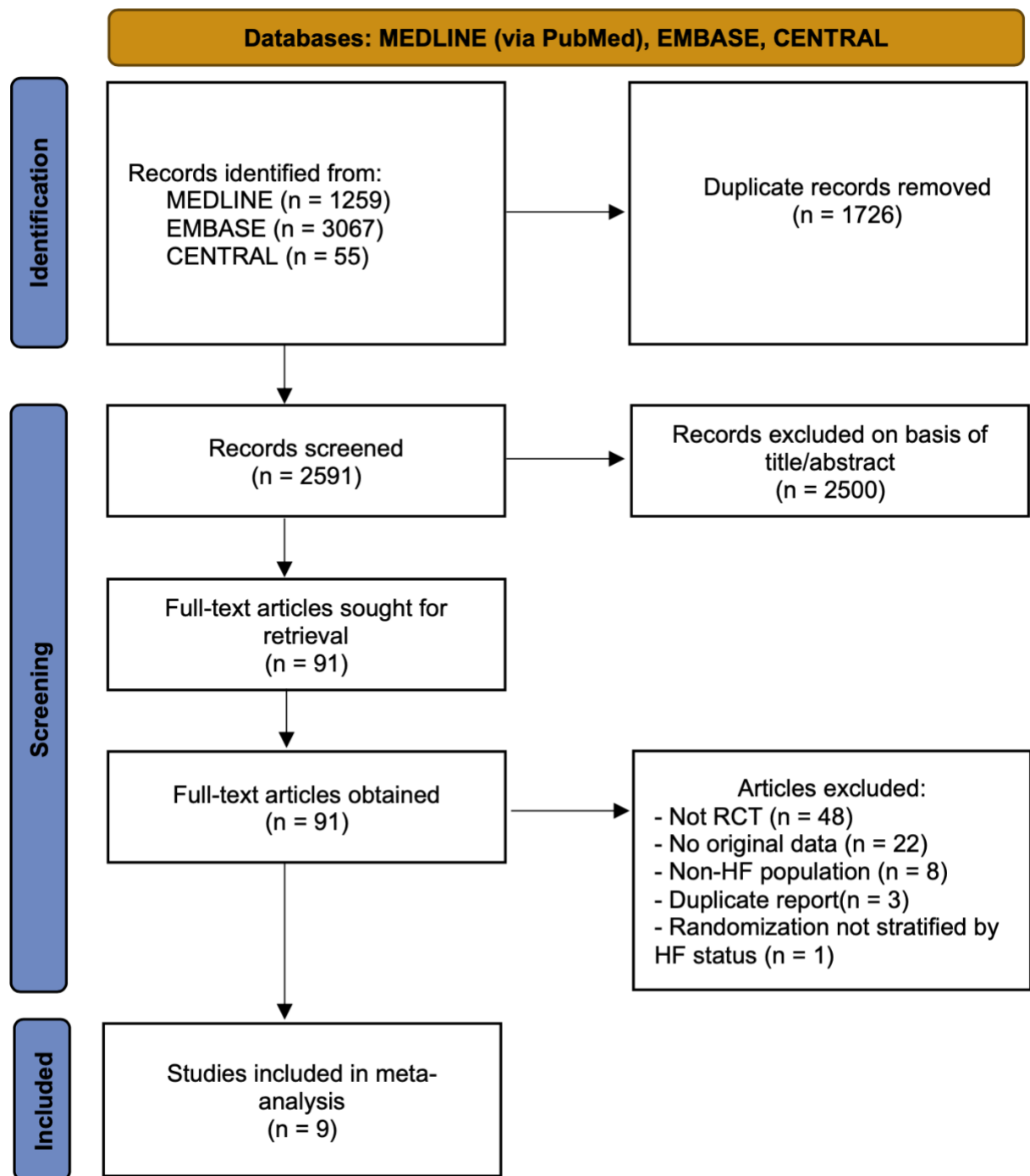

**Supplemental Figure 1:** PRISMA flowchart summarising study selection process

Supplement: Supplementary file 1 — Supplemental Figure S1. [file JOA3-39-129-s001.pdf]
