# Supplementary material for: Prognostic benefit of catheter ablation of atrial fibrillation in heart failure: An updated meta‐analysis of randomized controlled trials
Source: J Arrhythm. 2023 Jan 17;39(2):129–41. doi: 10.1002/joa3.12812 (PMC10068943; doi:10.1002/joa3.12812)
Supplement: Supplementary file 2 — Supplemental Figure S2. [file JOA3-39-129-s002.pdf]

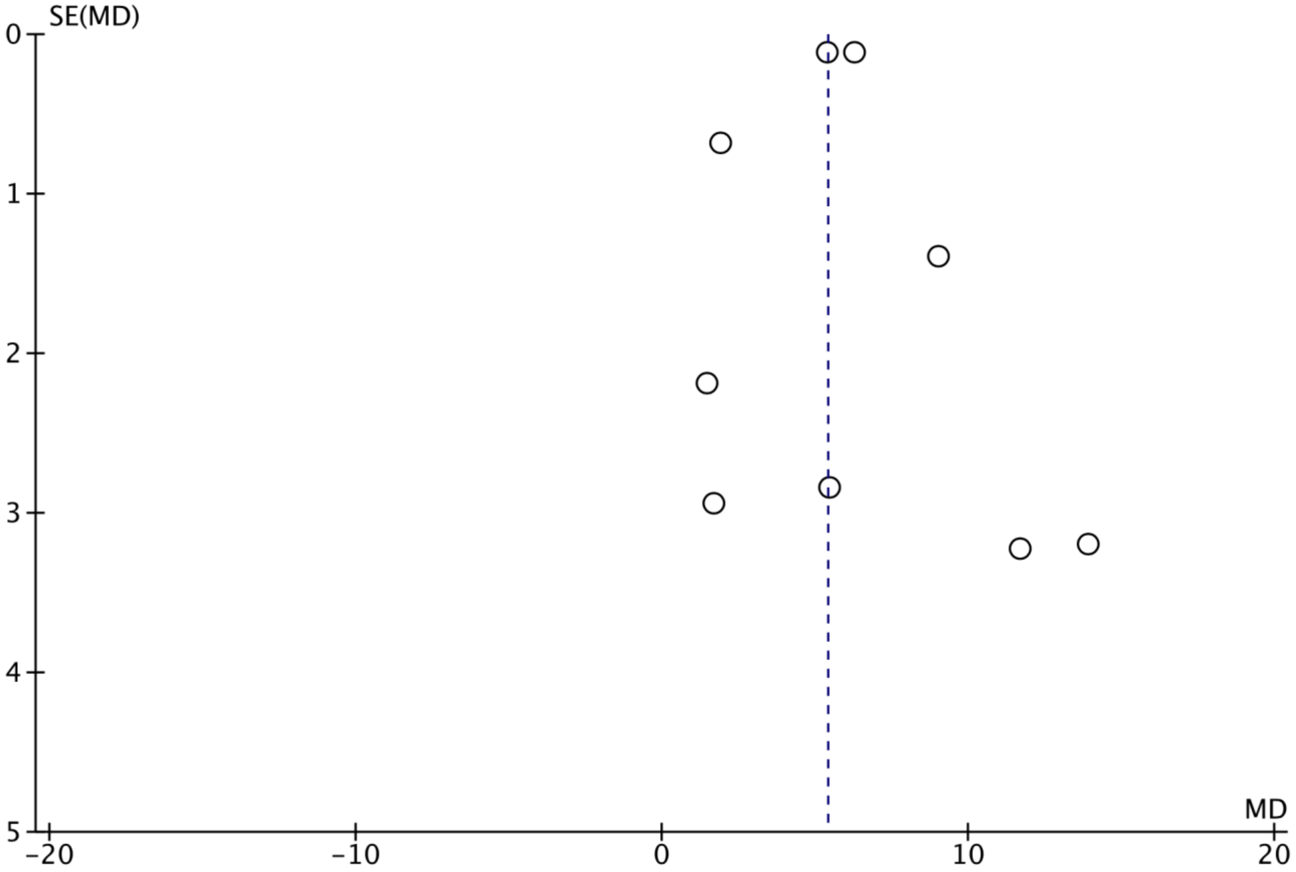

Supplemental Figure 2: Funnel plot displaying mean difference (MD) and standard error (SE) for primary endpoint of change in left ventricular ejection fraction
