# Supplementary material for: Prognostic benefit of catheter ablation of atrial fibrillation in heart failure: An updated meta‐analysis of randomized controlled trials
Source: J Arrhythm. 2023 Jan 17;39(2):129–41. doi: 10.1002/joa3.12812 (PMC10068943; doi:10.1002/joa3.12812)
Supplement: Supplementary file 3 — Supplemental Table S1. [file JOA3-39-129-s003.docx]

**Supplemental Table 1**. Quality assessment of included randomized controlled trials using Cochrane Risk of Bias Tool v2

| Study | Risk of Bias Domains | | | | | |
| --- | --- | --- | --- | --- | --- | --- |
|  | **Randomization** | **Protocol Deviations** | **Missing Data** | **Outcome Measurement** | **Selective Reporting** | **Other** |
| PABA-CHF 2008 | **Low risk** (computer generates sequences used for randomization) | **Low risk** (participants and investigators not blinded but no evidence this impacted protocol adherence) | **Low risk** (no significant loss to follow up) | **Low risk** (outcomes assessors blinded to treatment allocation) | **Low risk** (pre-specified endpoints reported) | 54% of screened patients excluded prior to randomization (reasons for exclusion given). |
| MacDonald 2011 | **Low risk** (centralized randomization using computer generated sequences) | **Low risk** (participants and investigators not blinded but no evidence this impacted protocol adherence) | **Low risk** (small losses to follow-up and ITT analysis used appropriately) | **Low risk** for primary endpoint of LVEF (blinded assessment)  **High risk** for subjective secondary endpoints (non-blinded assessors) | **Low risk** (pre-specified endpoints reported) | 89% of screened patients excluded prior to randomization (reasons for exclusion given). |
| ARC-HF 2013 | **Low risk** (randomization via computer generated sequences and appropriate allocation concealment) | **Low risk** (participants and investigators not blinded but no evidence this impacted protocol adherence) | **Low risk** (minimal loss to follow-up) | **Low risk** (outcomes assessors blinded to treatment allocation) | **Low risk** (pre-specified endpoints reported) | 49% of screened patients excluded prior to randomization (reasons for exclusion given). |
| CAMTAF 2014 | **Low risk** (random number generator used with appropriate allocation concealment) | **Low risk** (participants and investigators not blinded but no evidence this impacted protocol adherence) | **Low risk** (small losses to follow-up unlikely to be source of significant bias) | **Low risk** for primary endpoint of LVEF (centralized assessment)  **High risk** for subjective secondary endpoints (non-blinded assessors) | **Low risk** (pre-specified endpoints reported) | 87% of screened patients excluded prior to randomization (reasons for exclusion not given). |
| AATAC 2016 | **Low risk** (central computer generated blocks for randomization) | **Low risk** (participants and investigators not blinded but no evidence this impacted protocol adherence) | **Low risk (**minimal loss to follow-up and appropriate use of ITT analysis) | **Low risk** for primary endpoint of LVEF (blinded assessment)  **High risk** for subjective secondary endpoints (non-blinded assessors) | **Low risk** (pre-specified endpoints reported) | 77% of screened patients excluded prior to randomization (reasons for exclusion not given). |
| CAMERA-MRI 2017 | **Low risk** (centralized randomization by independent third party) | **Low risk** (participants and proceduralists not blinded but no evidence this impacted protocol adherence) | **Low risk (**minimal loss to follow-up and appropriate use of ITT analysis) | **Low risk** for primary endpoint (blinded assessment)  **High risk** for subjective secondary endpoints (non-blinded assessors) | **Low risk** (pre-specified endpoints reported) | 77% of screened patients excluded prior to randomization (reasons for exclusion given). |
| CASTLE-AF 2018 | **Low risk** (central computer generated sequences for randomization) | **High risk** (crossover of 16% and 10% in ablation and medical therapy arms, respectively; post randomization changes in inclusion/exclusion criteria) | **Low risk** (9% of randomized patients lost to follow-up for primary endpoint, statistically accounted for using both last observation carried forward and multiple imputation methods) | **Low risk** (all outcomes adjudicated by independent committee blinded to treatment allocation) | **Some concerns** (final analysis performed prior to reaching pre-specified event and enrolment targets) | 87% of screened patients excluded prior to randomization (reasons for exclusion given). |
| AMICA 2019 | **Low risk** (computer generated sequences for randomization) | **Low risk** (participants and investigators not blinded but no evidence this impacted protocol adherence, pre-specified imaging modality changed but unlikely to be a source of bias) | **High risk** for primary endpoint of LVEF (28% of patients with missing data at 1-year, sensitivity analyses performed using multiple imputation and prior measurement models) | **Low risk** for primary endpoint of LVEF (centralized assessment)  **High risk** for subjective secondary endpoints (non-blinded assessors) | **Low risk** (pre-specified endpoints reported) | Trial terminated early due to futility. No reporting on proportion of screened patients excluded prior to randomization. |
| RAFT-AF 2022 | **Low risk** (central web-based randomization with permuted blocks) | **Low risk** (participants and investigators not blinded but no evidence this impacted protocol adherence, minimal cross over) | **Low risk** (minimal loss to follow-up and appropriate use of ITT analysis) | **Low risk** (all outcomes adjudicated by committee blinded to treatment allocation) | **Low risk** (pre-specified endpoints reported) | Trial terminated early due to futility. No reporting on proportion of screened patients excluded prior to randomization. |

ITT, intention-to-treat; LVEF, left ventricular ejection fraction

**Supplemental Table 2**. Summary of meta-regression analyses for endpoint of change in left ventricular ejection fraction

| Covariates | Coefficient | 95% CI | P value |
| --- | --- | --- | --- |
| Publication Year | -0.14 | -0.48 to 0.20 | 0.42 |
| Mean Age (years) | -0.27 | -0.78 to 0.24 | 0.30 |
| Male (%) | 0.28 | -0.01 to 0.57 | 0.06 |
| Mean AF Duration (months) | -0.15 | -0.43 to 0.12 | 0.28 |
| Ischaemic cardiomyopathy (%) | -0.09 | -0.16 to -0.02 | 0.02 |
| NYHA III/IV (%) | -0.06 | -0.14 to 0.02 | 0.16 |
| Mean LA Diameter (mm) | -0.12 | -1.69 to 1.44 | 0.88 |
| Mean LVEF (%) | 0.20 | -0.08 to 0.48 | 0.16 |

AF, atrial fibrillation; CI, confidence intervals; LA, left atrium; LVEF, left ventricular ejection fraction; NYHA, New York Heart Association
